# Impact of Liquid Crystals in Active and Adaptive Optics

**DOI:** 10.3390/ma2020549

**Published:** 2009-05-12

**Authors:** Justo Arines

**Affiliations:** Departamento de Física Aplicada (Área de Óptica), Facultad de Ciencias / Universidad de Zaragoza, Zaragoza, Aragón, 50009, Spain; E-Mail: fajap@unizar.es; Tel. +34-976-762-849; Fax: +34-976-761-233

**Keywords:** liquid crystals, spatial light modulators, adaptive optics, wavefront correction

## Abstract

Active and dynamic modulation of light has been one of major contributions of liquid crystals to Optics. The spectrum of application range from signposting panels to high resolution imaging. The development of new materials is the key to continued progress in this field. To promote this we will present in this paper recent uses of liquid crystals as active or adaptive modulators of light. Besides, we will reflect on their current limitations. We expect with this to contribute to the progress in the field of liquid crystals and thus the development of new useful tools for Active and Adaptive Optics.

## 1. Introduction

Liquid Crystal (LC) materials have dramatically impacted our daily life in the last decades thanks to their optical properties. They can be found in a wide spectrum of applications: signpost panels, digital watches, calculators, cell phones, laptop displays… Nearly all the devices used in our daily life nowadays use a liquid crystal display. What is not so well known is the increasing use of liquid crystals in scientific optical applications: optical tweezers, beam shaping, tunable waveguides, digital holography, optical processors, wavefront sensing and correction, visual psychophysics, gray masks for photolithography, diffraction gratings... There are a huge variety of applications where the properties exhibited by the interaction of light with liquid crystals offer enormous design capabilities.

Which is the secret of Liquid Crystals? The answer lies the anisotropic organization of the molecules that provides a direction-dependant interaction with light. An electromagnetic field propagating through the material experiences different relative susceptibilities, depending on the relative orientation of liquid crystal directors and the direction of propagation and plane of vibration of the optical field. Additionally, due to its liquid like behavior, this relative orientation can be easily tuned by using electric, magnetic or optical fields [1,2,3,4,5,6], so anisotropy and tuning capability are the two main properties that make LCs so attractive.

In the preceding paragraph we spoke about relative susceptibility. This magnitude is related with two main properties: polarization and birefringence. The term polarization (P) refers to two different magnitudes. In one hand it describes the dipole moment per unit volume of a material that points in the same direction as the average molecular dipole moment. On the other hand it refers to the vibrational plane of an electromagnetic wave. The other term, birefringence, is an optical property of a material and refers to the anisotropic behavior of the refractive index [7]. This name comes from the splitting of the incident electromagnetic wave into two components, the ordinary and extraordinary rays. These rays experience different refractive indexes (*n_o_* and *n_e_)* and thus they propagate through the birefringent media with different velocities. At the end of the media one of the two electromagnetic components is delayed with respect to the other. If *n_e_ > n_o_* we have positive birefringence, otherwise it would be negative. Anisotropic materials can be classified in uniaxial or biaxial, depending on the eigenvalues of the refractive index (uniaxial presents two different values and biaxial three) [8,9,10].

Nearly all the applications of LCs in optics are related with their capability of inducing controllable amounts of retardation (or phase delay) and changes in the polarization state of the incident optical field [1,2]. In the next sections we will see the use of these principles in different optical applications.

Liquid Crystals are classified into two main families: thermotropic and lyotropic. Nearly all the LCs used in optical applications are thermotropic. Within this group the most outstanding phases are: nematic, smetic, and cholesteric (twisted nematic) [1,2,3,4,5,6].

Nematic liquid crystals present uniaxial positive birefringence. Smetic liquid crystals are normally uniaxial positive crystals, but they can also act as uniaxial negative or biaxial positive/negative crystals. Cholesteric liquid crystals (spontaneously twisted nematic) behave like uniaxial negative crystals. The main propertiy of this phase is its ability to rotate linearly polarized light. They present dichroism too. In these media one of the polarized components of light is much more absorbed than the other. This property is responsible for the typical iridescent color of cholesteric LCs [1,2,3,4].

Nematic, smetic and cholesteric LCs can be considered as waveplates with controllable continuous retardance. Within the smetic group there is an important phase that presents important optical characteristics, the chiral Smetic C. This subgroup is called Ferroelectric LC due to its permanent polarization without the need of an external electric field. They may be thought of as a waveplate with fixed retardance, but with an electrically controllable optical axis. They are faster than nematic ones and thus they are of interest for optical applications like optical switches or binary optics [11,12].

Lyotropic LCs also exhibit interesting optical properties [6]. As part of the the thermotropic group they can be uniaxial or biaxial negative and positive. Their main uses are restricted to the cosmetic and soap industry, but they have recently experienced a growing interest for optical applications [13,14,15,16,17,18,19,20].

Going back to birefringence, the existence of biaxial LCs has been theoretically proposed since the seventies [21]. Claims of achieving this in lyotropic phases date back as far from the eighties [15], but it took more than twenty years to achieve it in thermotropic ones [15,16,17,18,19,20,21,22,23,24,25,26,27]. The first such claims were demonstrated to be erroneous by the use of deuterium NMR spectroscopy [22]. It was only in 2004 that Madsen *et al.* and Acharya *et al.* provided firm evidence of the first biaxial nematic phase in thermotropic LCs [22,26]. Since then research on this topic has increased significantly. The potential of this kind of LC are enormous, considering the additional degree of freedom that it offers with respect to uniaxial LCs. Current proposals for the use of biaxial crystals concern their use as display compensators for improving the field of view or the color appearance [28]. Thus, the extension to the use of biaxial LC to these applications is only a question of time. Additionally, recent studies have shown the possibility of increasing the switching speed of LC valves by at least one order of magnitude with the use of biaxial LCs. This increase is obtained by rotating the LC along the molecules’ small axes instead of the longest ones [24,27].

Nonlinear optics has also benefited from liquid crystals. High second and third relative susceptibility values allow for nonlinear effects like second harmonic generation and intensity dependant refractive index change (Pockels and Kerr effects), photorefractive effect, molecular reorientation and thermal and density effects [1,2,12,29,30,31].

Thus, liquid crystals offer great flexibility in their optical behavior. Index of refraction, birefringent magnitude (nearly one order of magnitude higher than that of solids) and polarization properties can be selected by mixing different components. In the next sections we will present some examples related with the use of liquid crystals in different fields of optics. Then we will focus on their use in active and adaptive optics systems. Finally we present the conclusions.

## 2. Liquid Crystals in Optics

Optical properties of liquid crystals make them suitable for many applications [1,2,5,32]. Additional properties that have contributed to their expansion are: low cost, low power consumption, low weight, the fact they are non-mechanical devices, and easy addressability.

One of the first uses of liquid crystals in optical devices was as variable retarders and polarization rotators [33,34]. Nematic and smetic liquid crystals allow for a continuous variation of the retardance and polarization angle so they are the first options for these tasks. However these devices are too slow for certain applications (their switching speed is above 10 ms), so a lot of work has been devoted to increasing their time response by modifying the mechanical properties of the LC or by taking advantage of their nonlinear optical properties (for example by exploiting the Kerr effect in cyanobiphenyl molecules around their long axis in the smectic-A phase, which provides relaxation times of subpicosends [35]). A recent commercial result of the carrier for obtaining faster LC devices is a variable retarder for optical assemblies based on bulk-stabilized polymer liquid crystals, which have achieved switching speeds of less than 150 microseconds [36,12,27].

It is well known that ferroelectric LCs are faster (speeds over nanoseconds), but they present a big drawback, their binary behavior, so they provide bistable polarization orientation [11,12]. Considering switching applications they are the first option. However, there is a new trend in the search of faster LCs, the biaxial nematic phase. As said in previous paragraphs, their time response is one order of magnitude faster that uniaxial ones, while maintaining continuous tuning of optical retardance [26,27,28].

Most recent uses of liquid crystals are as tunable waveguides or active elements in integrated optical devices [37,38,39,40,41,42]. The guidance properties joined with their nonlinear response make LC suitable for integrated optical switchers, or logic gates [41,42]. The tunability of LC waveguides makes them extraordinarily useful for the study of optical propagation in complex structures. Recent studies have shown the possibility of using an array of channel waveguides in undoped nematic LCs to drive the system from one-dimensional bulk diffraction to discrete propagation and, even to nematicons (discrete spatial solitons in bulk nematic LC waveguides) [43]. A similar arrangement can be used to achieve multiband optical breathers [44]. Nonlinear optical responses of LCs can also be used for make optical routers by using the mode mixing approach, which consists of tuning the relative phase shift between the two propagation modes allowed by the waveguide in order to modulate their interference (see [45,46] for deeper analysis). Another example is the use of LCs in a tunable photonic-crystal waveguide Match-Zehnder interferometer where the LC changes the phase difference between the two arms of the interferometer (this device can be used as optical switcher or amplitude modulator in optical circuits [37]). Three-dimensional data storage, or holographic storage is another field where LCs play an important role. Data recording with density of 204.8 Gbits/cm^3^ was recently achieved using the two-photon excitation technique [47].

## 3. Spatial Light Modulators, Active and Adaptive Optics

There is one application of liquid crystals that stands out above the others, their use in Spatial Light Modulators (SLMs). A SLM is a device that modulates the spatial characteristics of light (for example LC displays). Depending on its configuration it can modulate the amplitude, the phase or both magnitudes of an impinging optical field. Liquid Crystal Spatial Light Modulators (LCSLMs) are classified into two main categories, depending on how they are addressed. If the electric field is electrically controlled by a matrix of pixels, then it is an electrically-addressed LCSLM. If an optical field is used to control the SLM, then it is called an analogue-addressed or optically-addressed LCSLM [48].

Liquid crystal display technology employs electrically driven LCSLMs. The commercial applications of this technology have increased the research efforts and the investment in new materials. Contrast ratio, viewing angle, polarization sensitivity, electronic consumption, temperature stability, operating speed and computer configuration are some of the characteristics of the new devices. The main disadvantages of these LCSLMs are: polarization sensitivity, time response, and above all pixelization, which reduces the fill factor and induces light diffraction. In contrast, optically-addressed LCSLMs were developed for scientific applications in order to create an SLM free of the diffraction effect induced by the pixel matrix used in the electrically driven LCSLMs. However these are less flexible in their configuration and use.

Scientific research has benefited from the developments in LC displays. Application of LCSLMs to photolithography, optical tweezers, optical processing, beam shaping and active and adaptive optics are some of the fields that have experienced a big increase due to the use of SLMs in the last decade [49,50,51,52].

Photolithography is a very important technique for manufacturing microelectronic and microoptical devices. It consists of exposing a photoreactive material to light through an amplitude mask. This mask can be binary or continuous. The SLM offers the flexibility of varying the mask type and its spatial distribution just by sending different images to them [49].

An optical tweezer is a device used to trap and guide particles or cells [50]. As photolithography the SLM is used for modulating the spatial distribution of the irradiance of the focal distribution of a light beam by changing the spatial distribution of the amplitude and or phase of the optical field at the exit pupil of the system [51]. This procedure is also used for beam shaping [52].

### 3.1. Active and Adaptive Optics

Active and Adaptive Optics are two technologies characterized by the use of a configurable electro-optical device that can be adapted for the correction of the wavefront degradation [53,54,55,56]. These techniques were first used for non military purposes for the correction of the atmospheric turbulence degradation in order to obtain high resolution astronomic images. The main difference between active and adaptive optics is their time response. While the term “active optics” is used when the correction is adjusted at low frequency and the changes in the degradation function are slow, the adaptive term is used in cases for which degradation changes continuously and the correction must be quickly adapted. Thus, in adaptive optics systems (AOs) we need not only the electro-optical device for compensation, but also a wavefront sensor.

Traditionally AO used deformable mirrors for accomplishing the wavefront compensation. These devices work by introducing optical path differences between different parts of the wavefront. Two kind of approaches has been used: segmented and membrane deformable mirrors. Segmented deformable mirrors can be of bigger aperture than membrane ones, and are easy to control. However they present diffraction effects for the individual segmented edges and difficulty for achieving inter-segment alignment. On the other hand membrane deformable mirrors are difficult to control, and present smaller aperture, but they avoid the diffraction effect [53,55].

Liquid crystal spatial light modulators operating in pure phase modulation mode have been used in the last twenty years as an alternative to deformable mirrors [54,57,58,59,60,61,62,63,64,65,66,67,68,69]. These devices introduce controlled amounts of retardance at different parts of the wavefront. Their initial limitation concerning speed, polarization sensitivity, retardance range, and diffraction effect reduced their expansion. However they have been overcome nowadays by using different approaches. For example, polarization sensitivity can be avoided by using a quarter wave plate and a mirror as proposed by Love [69] or by using a device with two active LC layers that are orthogonal to each other [70]. Speed can be increased by using the double frequency scheme [71]. The diffraction effect is not present in optical-addressed LCSLMs [48,72]. The use of LCSLMs for astronomical proposes is still a challenging question. Management of small amounts of polarized light of different wavelengths is still better accomplished with deformable mirrors, although the response of LCSLMs has been greatly increased.

The future of LCSLMs is not restricted to astronomical applications. In fact, their main uses are out of that field. The active and adaptive optics philosophy has been applied in different fields of research. Clinical applications for early diagnosis and patient monitoring as well as basic studies on physiological optics and vision science have also benefited in the last few years [73,74,75,76,77,78,79,80,81]. Low price, easy handling and easy control of LCSLMs are the main reasons that have encouraged different research groups to initiate lines of research based on SLMs that were initially restricted to well funded groups capable of acquiring the expensive deformable mirrors. Additionally, the flexibility in the design of the coded phase allowed to uses the same LCSLMs to perform different tasks. The same device can be used to code wavefront sensors, amplitude masks, or wavefront modulators, different optical elements that are important parts of complex optical assemblies.

### 3.2. Low Cost Active and Adaptive Optics

Within the LCSLM market there are some devices especially manufactured for wavefront sensing and control, like those manufactured by Hamamatsu Photonics, Meadowlark Optics or Holoeye. These devices are continuously evolving, increasing the sampling rate, wavelength range, time response, and amount of retardance, among other features. They are not as expensive as deformable mirrors, but are not especially cheap. Another important user of LCSLMs is the video projector industry. Although the LCSLMs used in that devices are specially designed for providing high quality images, they can be also used for scientific applications. Their specifications are not as good as those built for wavefront applications, but they satisfy the minimum necessary requirements. In addition to this, their price is continuously decreasing following the trends of the audiovisual market. Besides, these VPTNLCs are robust and use the same interconnection hardware and software as video projectors, which increases their flexibility and simplicity of control.

**Figure 1:**
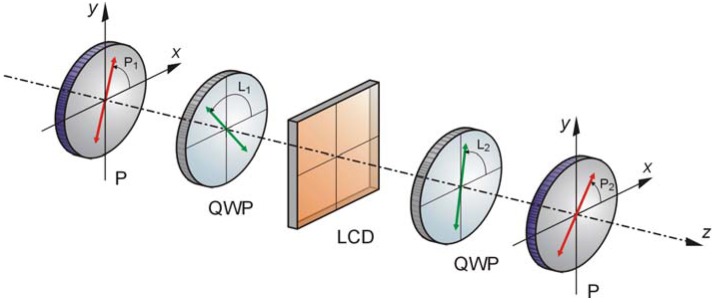
Phase-only modulation experimental setup with a VPTNLC. P: polarizer; QWP: a quarter-wave plate. In the diagram, the x-axis coincides with the input molecular director of the liquid crystal cell. P1 and P2 are, respectively, the orientation of the polarizer and the analyzer. L1 and L2 are the angles of the slow axis of the quarter-wave plates with respect to x-axis.

One of the main limitations of LCSLMs, and off course VPTNLCs, is the retardation range, normally ranging under one wavelength. Optical researchers had overcome this limitation by projecting a wrapped image instead of the continuous one in the same way as the diffractive optics elements (see [7] and [80] for more details). In a recent study our group, in collaboration with researchers from the University Jaume I in Castellón (Spain), used a VPTNLC with a four level encoding scheme in an adaptive optics system used to compensate aberrations like those presented by human eyes [80], which in general are above one wavelength of magnitude. The authors used in that study an optical arrangement for operating with the VPTNLC in a pure phase modulation mode. See Figure 1 for details.

**Figure 2:**
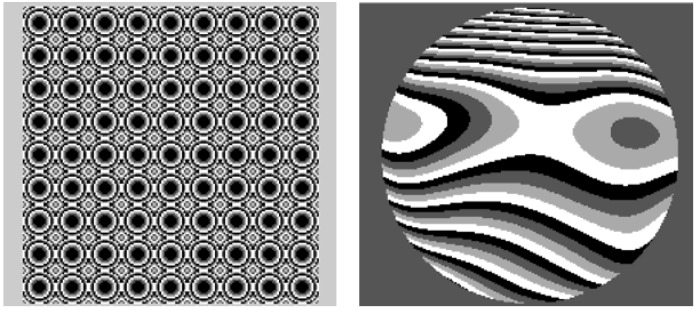
Grayscale representation of the four-level VPTNLC patterns: (left) for generating a 9 x 9 diffractive microlens array; (right) for compensating the aberration produced by an artificial eye.

That system presented an additional characteristic, the same VPTNLC was used for the measurement of the wavefront aberration and its compensation. This means that we encoded in the VPTNLC a microlens array (sampling element used in Hartmann-Shack wavefront sensors [53,54,55]) for measuring the distorted wavefront and then the aberration compensation. These two steps were repeated in a close-loop in order to correct the wavefront aberration. In Figure 2 we show the two images sent to the VPTNLC.

In the next figure we show the system operating in close-loop. We can see the focal spots of the microlens array, the four level encoded compensation, and degraded and restored image.

**Figure 3:**
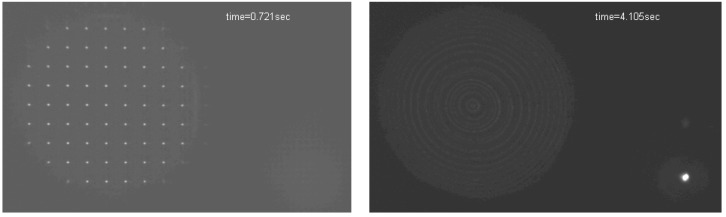
A real time video of the performance of the system when compensating continuously varying amounts of defocus. See the supplementary video for observing the system operating in close-loop.

At this point we want to remark one of the characteristic of VPTNLCs, flexibility. We can use the same VPTNLC cell for measuring and compensating the wavefront aberration. Additionally we can align by software the sampling array and the compensation element with the exit pupil of the optical system easily by displacing the projection coordinates of the images, alignment with a precision of one pixel.

## 4. Final Remarks

Liquid crystal materials offer a variety of properties that make them attractive for a lot of applications. The secret of their success is their anisotropic molecule organization and the capability of change their orientation by the application of controlled electric, magnetic or optic fields.

Their anisotropy is responsible for their most important optical properties. Birefringence, polarization rotation and significant values of 2^nd^ and 3^th^ order relative susceptibilities make liquid crystals one of the most interesting materials for the future optics.

Thermotropic liquid crystals are the most used for optical applications and among them the nematic and twisted nematic phases are the preferred ones. The main advantage of these phases over smetic ones is their capability of continuous variation of the induced retardation. However in applications like optical switching were to states (on/off) are desired, ferroelectric liquid crystals are the first option due to their fast time response.

In this paper we have shown the variety of applications that liquid crystals had found in optics. Variable retarders and polarizers, optical switchers and spatial light modulators are some of the most outstanding ones.

Special emphasis has to be made with respect to spatial light modulators. Their use for phase and/or amplitude modulation has experienced an enormous increase in the last decade, mainly due to their low cost, easy addressability and flexibility. Different applications have been found for SLM, among them Active and Adaptive Optics for astronomical and non astronomical tasks must be remarked. The high potential of LC in this field has not been reached yet. The application of this technique for the manipulation of light offers a lot of applications that still remains to be proposed. Light traps, pholitography, digital holography, optical tweezers and tunable aberrated lenses, are part of the present. Vision sciences has also benefited from VPTNLCs. Visual tests devoted to determine the influence of controlled amounts of aberration on vision [73], the use of adaptive wireless intraocular LC lens that provides an accommodation amplitude of 2.5 diopters [75], and high resolution imaging of the retina are some of the applications in this field, [82,83].

The optical demands that fall on liquid crystal science encourage the research of new materials. Polymer stabilized liquid crystals [36] or LC doping with dichroic dye dopants [84] or nanoparticles [85], and biaxial phases [25,26] are some of the lines of research that are now being exploited. These studies are mainly devoted to increase the projection devices. However as has been shown in this paper, there are other specific applications that can take advantage of those efforts.

Liquid crystal science is one of the most interdisciplinary fields that exist today. The variety of applications found for liquid crystals, urge the collaboration between scientists of different disciplines. In this way, we hope that the presentation of some of the most relevant applications of liquid crystals in optics had contributed to the development of new liquid crystals useful for optical applications.
